# Clinical evaluation of subepithelial connective tissue graft and guided tissue regeneration for treatment of Miller’s class 1 gingival recession (comparative, split mouth, six months study)

**DOI:** 10.4317/jced.51302

**Published:** 2014-07-01

**Authors:** Sakshee-R. Trivedi, Neeta-V. Bhavsar, Kirti Dulani, Rahul Trivedi

**Affiliations:** 1 Senior Lecturer. Department of Periodontology and Implantology, Government Dental College and Hospital, Asarwa, Ahmedabad, Gujarat, India; 2 Professor and Head. Department of Periodontology and Implantology, Government Dental College and Hospital, Asarwa, Ahmedabad, Gujarat, India; 3 Senior Lecturer. Department of Periodontology and Implantology, Government Dental College and Hospital, Asarwa, Ahmedabad, Gujarat, India; 4 Senior Lecturer. Department of Orthodontics and Dentofacial Orthopedics, Government Dental College and Hospital, Asarwa, Ahmedabad, 380016, Gujarat, India

## Abstract

Objectives: The present study aims to clinically compare and evaluate subepithelial connective tissue graft and the GTR based root coverage in treatment of Miller’s Class I gingival recession. Study Design: 30 patients with at least one pair of Miller’s Class I gingival recession were treated either with Subepithelial connective tissue graft (Group A) or Guided tissue regeneration (Group B). Clinical parameters monitored included recession RD, width of keratinized gingiva (KG), probing depth (PD), clinical attachment level (CAL), attached gingiva (AG), residual probing depth (RPD) and % of Root coverage(%RC). Measurements were taken at baseline, three months and six months. A standard surgical procedure was used for both Group A and Group B. Data were recorded and statistical analysis was done for both intergroup and intragroup. Results: At end of six months % RC obtained were 84.47% (Group A) and 81.67% (Group B). Both treatments resulted in statistically significant improvement in clinical parameters. When compared, no statistically significant difference was found between both groups except in RPD, where it was significantly greater in Group A. Conclusions: GTR technique has advantages over subepithelial connective tissue graft for shallow Miller’s Class I defects and this procedure can be used to avoid patient discomfort and reduce treatment time.

** Key words:**Collagen membrane, comparative split mouth study, gingival recession, subepithelial connective tissue graft, guided tissue regeneration (GTR).

## Introduction

Nowadays, patients have become increasingly aware of the gingival recession and its unaesthetic features. The exposure of cementum and dentin leading to dentinal hypersensitivity becomes a constant discomforting factor to patients in everyday life. Patients present with complaints of dentinal sensitivity in areas of recession even where the defect is shallow. Such defects associated with or without abrasion cavities, increase the susceptibility to root caries (1).

With changing paradigms in dentistry, aesthetic dentistry has evolved as an interdisciplinary approach treating multitude of problems and meeting patients’ expectations. Amongst various techniques that have been described for the treatment of gingival recession, their efficacy and predictability are important parameters for both the patient and clinician. From the patient’s perspective, an attempt to reduce the number of surgeries and cost factor must be taken into consideration.

Subepithelial connective tissue graft [SCTG] technique, initially described by Langer & Langer (2) is a standard technique with predictable and reproducible results. It yields 84.84% (3) to 96% (4) in areas ≥ 3 mm and 80% to 100% (5) results in areas with ≤ 3 mm of recession depth.

Recently, use of collagen membranes in Guided tissue regeneration [GTR] for root coverage has also shown promising results (1,3,5,6). Bilayered collagen membranes provide sufficient space below the flap which pro-motes new connective tissue formation and effectively inhibits epithelial migration (7).They are readily absorbed and hence eliminate need for second surgery or a graft harvest site (3). Like subepithelial connective tissue, the bilayered membrane may act as a scaffold and increase the recipient site tissue thickness (8). GTR based recession coverage procedures have demonstrated results comparable to that obtained by SCTG (1,3,5,6).

The purpose of this study was to compare and evaluate the GTR based root coverage using bioabsorbable bilayer collagen membrane [#: ProGide ™, Bi-textured resorbable barrier, Equinox] and SCTG based root coverage procedure for treatment of shallow Miller’s Class I recession defects. An attempt has been made to evaluate the utility of GTR based root coverage as compared to subepithelial connective tissue with respect to patient acceptation and aesthetic results.

## Material and Methods

The study was a clinical, comparative, split mouth, randomized control trial with a time period of six months. An Ethical Committee approval was obtained before commencement of the study. The sample subjects were selected randomly from amongst the patients referred to the Department of Periodontology and Implantology, Government Dental College and Hospital, Ahmedabad, for complaints associated with gingival recession like unaesthetic looks & dentinal hypersensitivity, in maxillary and mandibular anterior teeth and premolars. A total of 30 pairs of defects were treated. Patient inclusion criteria was systemically healthy adults with realistic expectations and age up to 50 years, non pregnant, non-smokers, with no history of antibiotic treatment within three months from the time of commencement of study, at least one pair of comparable Miller’s class I recession defects in anterior teeth and premolars of maxillary and mandibular arches, good oral hygiene and sufficient palatal donor tissue. All selected subjects were explained nature of the study and a written consent was obtained on a consent form approved by the Ethical Committee. Initial therapy consisted predominantly of oral hygiene instructions. Inappropriate or faulty oral hygiene maintenance techniques were rectified. Patients were instructed to adopt Modified Stillman’s method for cleaning in areas with gingival recession. Scaling and root planing was done prior to surgical therapy. Any existing trauma from occlusion was eliminated. An appointment for the surgical procedure generally was arranged 10 days after the initial procedure. At the pre-operative examination, the teeth demonstrating recession were examined with respect to soft tissue parameters. Most paired defects were treated in the same surgical session or in two consecutive appointments. Randomization for Subepithelial Connective Tissue graft [Group A] and Guided tissue regeneration based root coverage [Group B] was performed by coin toss at the beginning of the study. The right side was selected for Group A and left side for Group B.

**Figure 1:**
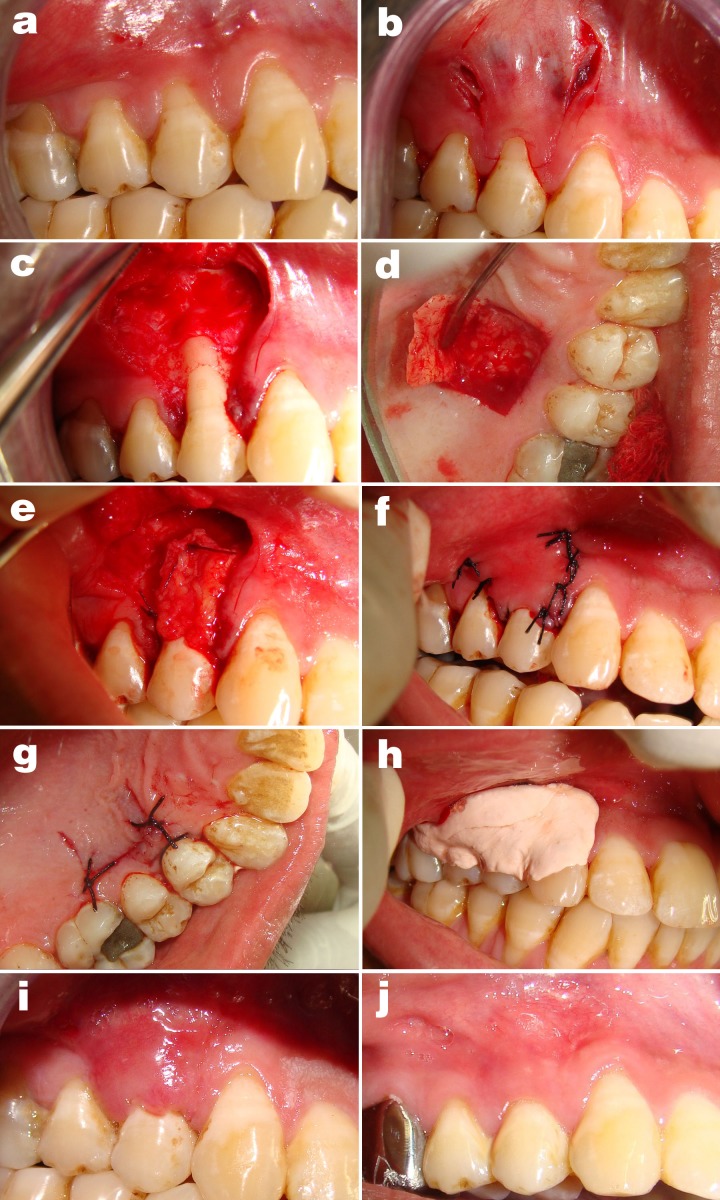
Subepithelial connective tissue graft (Group A). a. Pre-operative gingival recession; b. Incision; c. Reflection of flap; d. Preparation of the palatal donor site; e. Graft sutured; f. Coronally positioned flap covering the graft sutured without tension; g. without tension; h. Palatal donor site closed; i. Periodontal pack on the treated site; j. Healing after 10 days; k. Healing after six months and Gingivoplasty.

**Figure 2:**
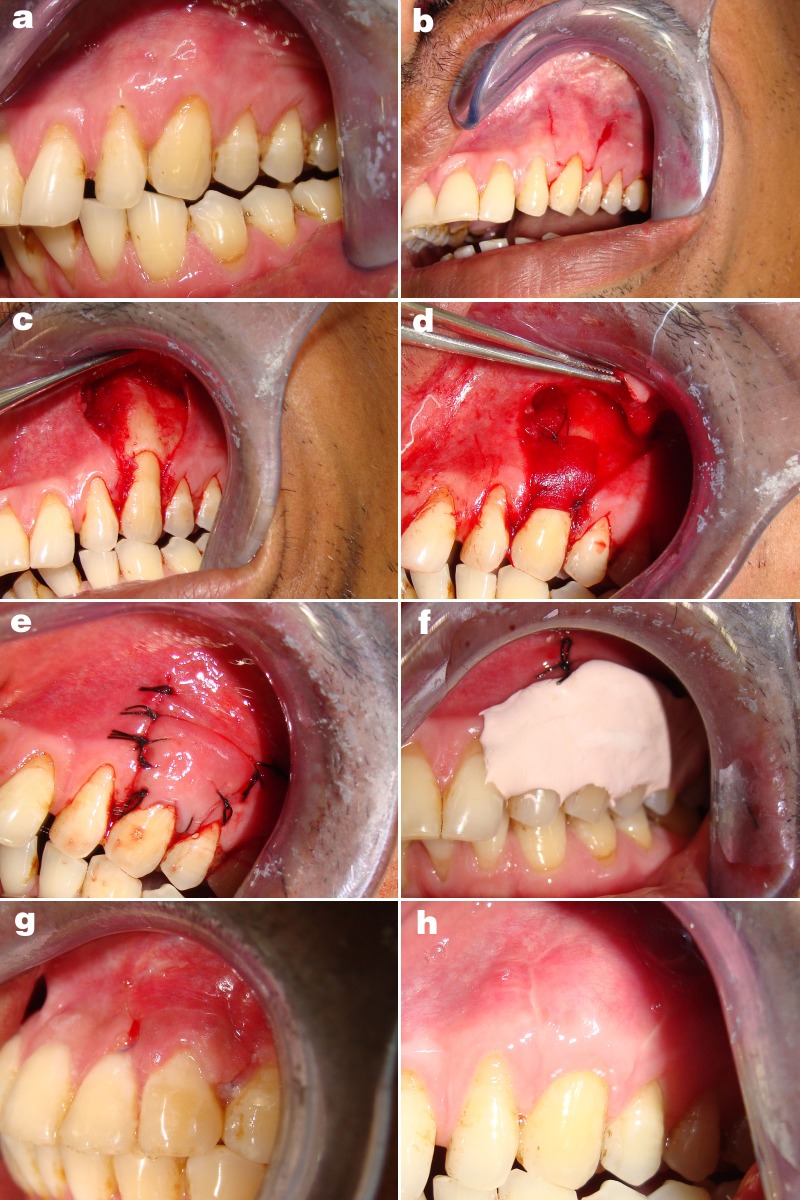
Guided tissue regeneration based root coverage (Group B). a. Pre- operative gingival recession; b. Incision; c. Reflection of flap; d.Membrane sutured; e.Coronally positioned flap covering the membrane sutured without tension; f. Periodontal pack on the treated site; g. Healing after 10 days; h. Healing after six months.

- Clinical Parameters: Soft Tissue Parameters: [Measured At The Selected Sites]: An acrylic stent that acted as a fixed point at the level of cemento-enamel junction to make accurate measurements of root exposure with the help of UNC- 15 probe [@: InSci, Equinox], both pre-operatively and post-operatively was used. The measurements were taken at the midfacial aspect of the tooth. Parameters were: Gingival Recession Depth [RD]; Width Of Keratinized Gingiva [KG]; Clinical Attachment level: [CAL] ; Probing depth [PD] ; Residual Probing Depth [RPD]; Attached gingiva [AG]; Percentage of root coverage [ %RC] = [Post Operative RD – Preoperative RD X 100%]/Preoperative RD. RD, KG were recorded pre-operatively and post- operatively at 10 days ,one month, three months and six months. CAL, PD, AG were recorded pre-operatively and post-operatively at three months and six months. %RC was calculated at six months post operative.

- Preparation of the recipient sites: (Fig. 1,2) 

The surgical area was prepared with adequate local anesthesia, using 2% Lignocaine incision in a mesio-distal direction, extending into the adjacent interdental area slightly coronal to the tooth’s CEJ. Care was taken not to involve the entire papilla. An intrasulcular incision was made to join the horizontal incision. Two apically divergent vertical incisions placed at each end of the horizontal incisions extending apically into the alveolar mucosa were placed. A full thickness flap was elevated and 2 mm color of bone was exposed, after which a partial thickness flap was elevated to the mucogingival junction and a partial thickness dissection was done into the alveolar mucosa to allow for the release and coronal positioning of the flap. The intact papillae mesial and distal to the recession site were de-epithilized. The exposed, affected root surface was scaled and planed. After all site preparation was completed, the measurements were recorded for the size of the membrane and the graft.

- Preparation of the donor site and graft retrieval: (Fig. 1)

A second surgical site was created on the palate. The subepithelial connective tissue connective tissue was retrieved using “The Trap Door” Technique: Nelson S.W [1987] (9).

- Placement and suturing.

The graft (Fig.1) and the membrane# (Fig. 2) were shaped to fit their respective sites. The graft and membrane# extended laterally and apically beyond the bony margins of the dehiscence/ recession and covered by the host flap. The subepithelial connective tissue graft and the membrane were fixed in place with coronal tacking with sling sutures using a resorbable 5-0 sutures [†: 5-0 Vicryl™ [polyglycolic braided] J490 [Ethicon]] at the level of CEJ. The flap was then coronally positioned to cover the membrane and the graft without tension and secured at the position by a sling suture with 4-0 silk suture [‡:4-0 Mersilk™ [Braided silk black] NW 5050 Ethicon Johnson & Johnson LTD, Baddi, H.P.-173205 INDIA] and atraumatic needle, over the de-epithilized papillae. Loop sutures were used for vertical incision. A protective sterile foil was place over the surgical site and periodontal pack [§: COE PAK™ Periodontal dressing Regular set, GC America INC. Alsip, IL 60803 U.S.A] [non-eugenol periodontal dressing] (Fig. 1,2) was given. Post surgical instructions were given. Antibiotic & Anti-inflammatory drugs were prescribed. Sutures were removed after 10 days.

- Follow Up Care.

Patients were seen at 10 days (Fig. 1,2) one month, three months, and six months (Fig. 1,2). After removing periodontal dressing, brushing was avoided at the treated site. Instead, cotton pellet was used to clean and slightly comb the area an apical to coronal direction for the next 4 weeks. Data was recorded at every visit. Reinfor-cement of oral hygiene instruction was also performed. At the end of six months, each patient was evaluated for queries related to their experience of each surgical procedure ( Table 1).

**Table 1:**
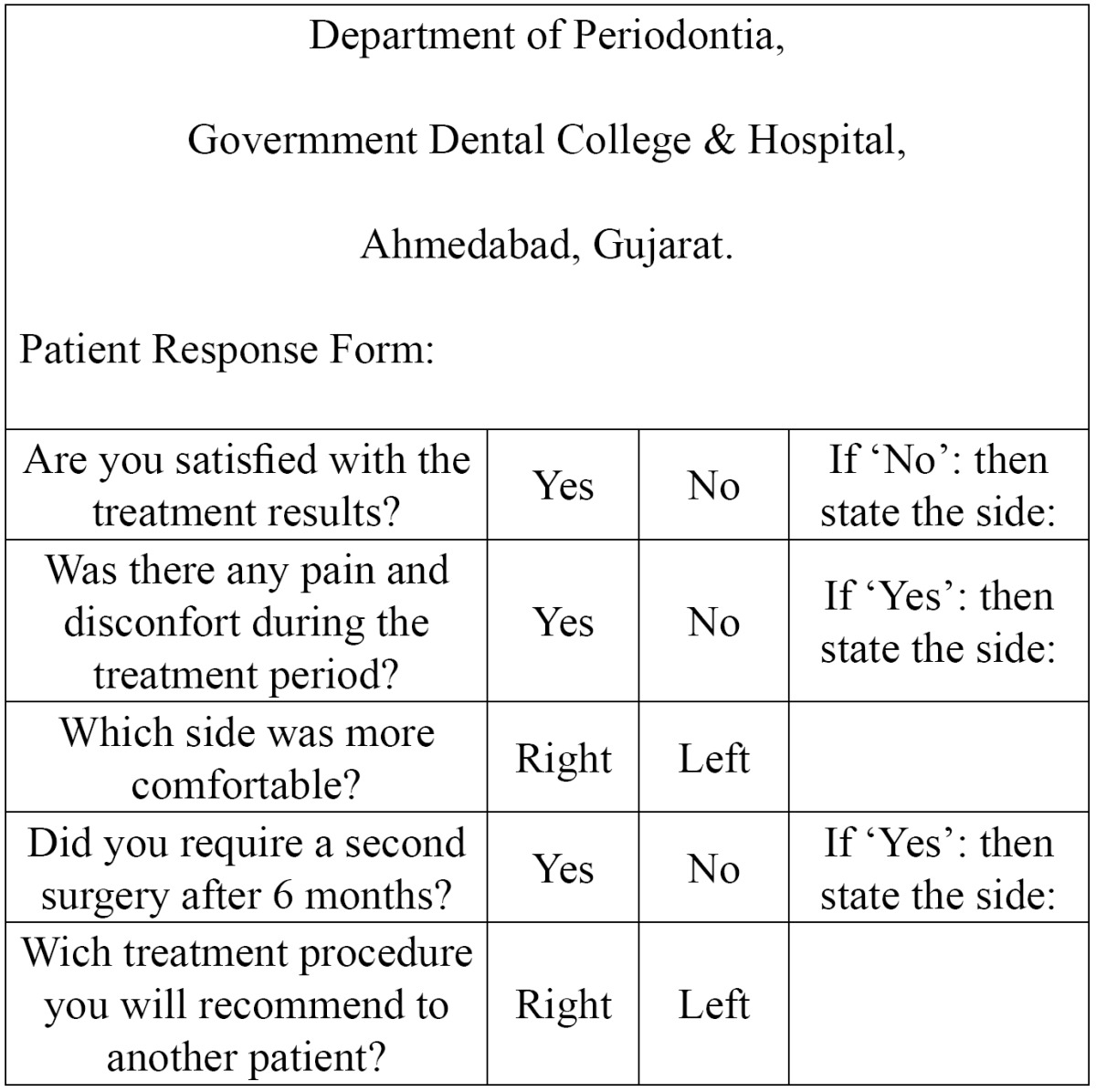
Patient response form.

- Statistical methodology:

The data gathered from the present study was tabulated and analyzed using suitable techniques. Data were reported as Mean ± Standard Deviation [mean ± std] in millimeters [mm]. To study the effect overtime within groups the paired “t” test was used. The changes in average RD, KG values at one month, three months and six months from pre-operative values were tested. The changes in AG, CAL and RPD values at three months and six months from pre-operative values were tested. Further the average change from pre-operative to six months of the above mentioned parameters were compared in between groups to see the difference using Student “t” test. The “t” test values were compared with table values to show the level of significance.

## Results

30 pairs of Miller’s class I recession defects were treated with either SCTG [Group A] or GTR [Group B]. No case was reported for any post surgical complication or exposure of membrane. On the analysis of the data, there was improvement in all parameters ( Table 2) and no difference between the groups at baseline for all the clinical parameters ( Table 3).

**Table 2:**
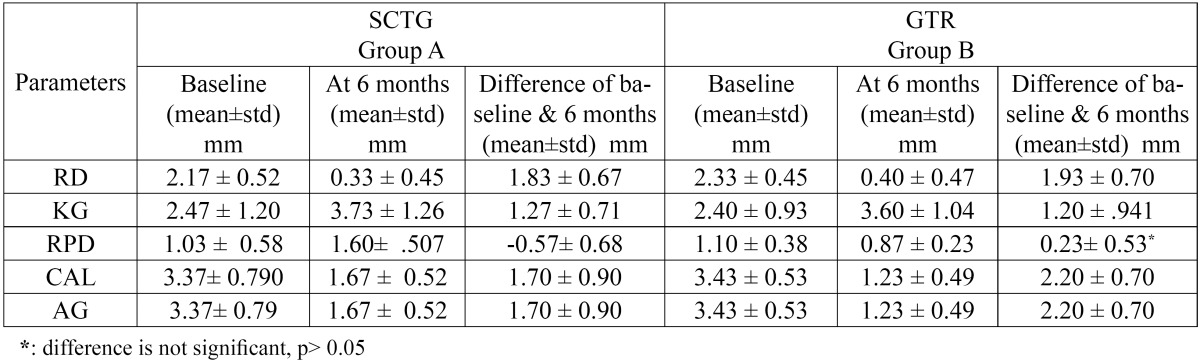
Clinical parameters at baseline and 6 months.

**Table 3:**
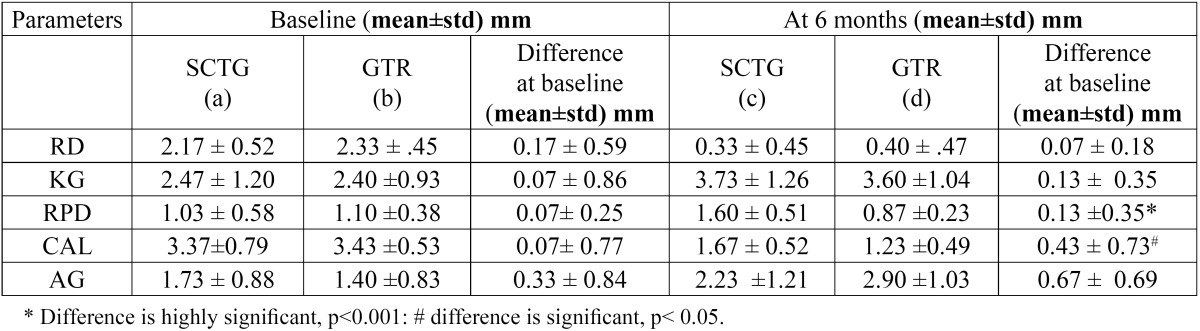
Comparison of clinical parameters of Group A and Group B ant Baseline and at Six Months.

The mean reduction of RD at six months post-operative in Group A was 1.83 ± 0.67 mm; p value <0.001 ( Table 2). The %RC obtained at sis months post-operatively was 84.47 ± 21.07 % ( Table 4). The mean reduction of RD at six months post-operative in Group B was 1.93 ± 0.70 mm; [p value <0.001] ( Table 2). The %RC obtained at six month post-operatively was 81.67 ± 22.31 % ( Table 4). After six months of treatment, there was no statistically significant difference between the reduction of RD between both groups [0.07 ± 0.18 mm; p value>0.05] ( Table 3). The difference between the %RC in the present study was not significant [2.80 ± 7.49; p value >0.05] between both groups ( Table 4). The mean increase in KG at six months post-operative in Group A was 1.27 ± 0.71 mm; [p value <0.001] and in Group B was 1.20 ± 0.941 mm; [p value <0.001] ( Table 2). After six months of treatment, there was no statistically significant difference between the mean increase in KG between both groups [0.13 ± 0.35 mm; p value>0.05] ( Table 3). There is highly significant increase in RPD at six months postoperatively [1.60 ± 0.507 mm] in Group A ( Table 2). There is decrease in postoperative mean RPD in Group B, however the difference is not significant [0.23 ± 0.53 mm; p value>0.05] ( Table 2). At six months after treatment, there was highly significant difference between groups [0.13 ± 0.35; p value <0.001] ( Table 3). The gain in CAL, for Group A is 1.70 ± 0.90 mm; p value <0.001 and for Group B is 2.20 ± 0.70 mm; p value <0.001 ( Table 2). When both the treatments were compared at six months for the difference in CAL, the difference was found highly significant with more gain in clinical attachment in Group B [0.43 ± 0.73 mm; p value <0.001] ( Table 3). At the end of six months, there is an increase in AG in both groups [Group A: 0.50 ± 0.85 mm; p value <0.05]. Group B: 1.50 ± 0.76 mm; p value <0.001 ( Table 2). When both the treatments were compared at six months for the difference in AG, the difference was found significant with more gain of attached gingiva in Group B [0.67 ± 0.69 mm; p value <0.05] ( Table 3). After six months all the patient response forms were collec-ted and data was grouped ( Table 5).

**Table 4:**
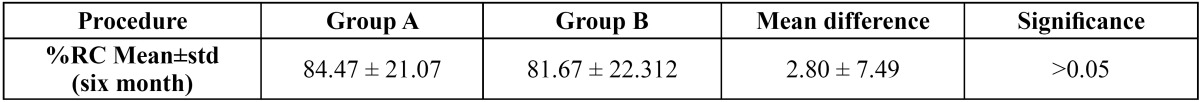
Comparison of mean change of % of root coverage at six months between Group A and Group B.

**Table 5:**
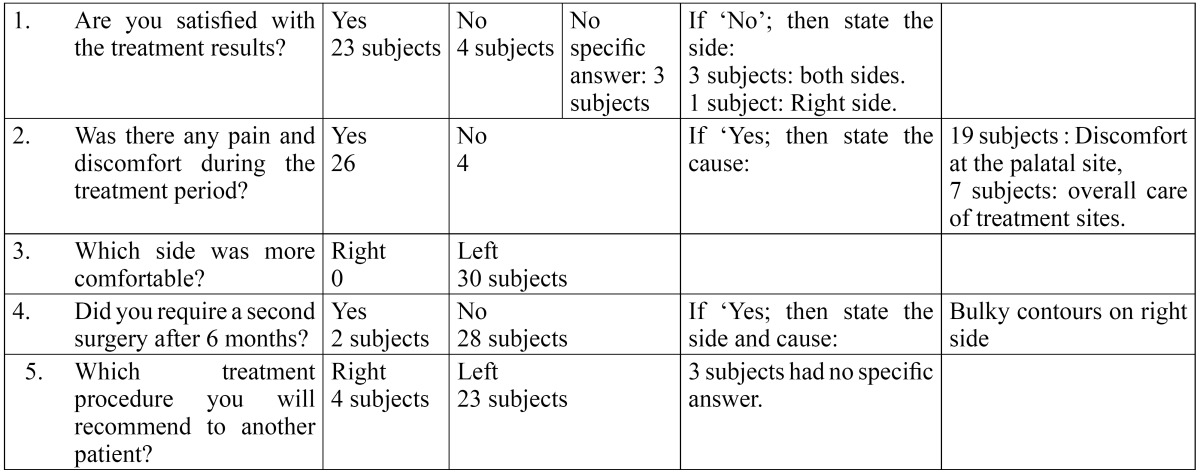
Data from Patient Response Form.

## Discussion

The present study was designed as randomized split mouth study, in order to eliminate patient response bias and patient related factors like post operative care, healing and oral hygiene maintenance. 30 patients were selected such that, at baseline no significant difference was present among the clinical parameters recorded. For both Groups A and B, same surgical technique was used to prepare recipient site so as to standardize comparisons. All patients completed study without any uneventful healing during initial and later phase of treatment. This can be attributed to strict surgical protocol, aseptic conditions and patient co-operation to follow post surgical instructions.

Bilayered collagen membranes are known to effectively inhibit epithelial migration and provide sufficient space for appropriate cells [e.g. PDL cells, bone cells] to repopulate the area (10). No case of exposure of membrane was reported. This could be due to the use of bilayered collagen membrane (3) and properties of collagen to augment flap thickness by providing a collagenous scaffold (1). One of the important factors increasing the risk for gingival recession may be a thin and delicate marginal tissue covering a non-vascularized root surface (11). In case of surgical coverage of denuded root surface, it might therefore be desirable to increase the dimensions of the tissue i.e. width and thickness of keratinzed gingiva for preventive reasons (11). Subepithelial connective tissue graft offers similar advantage of increasing recipient site thickness. Some site may require a gingivoplasty procedure to achieve final form and contour. Two cases in the present study required gingivoplasty.

An important criterion for success of GTR is the post surgical stability of the coronally advance flap [CAF] that completely covers the membrane. At least 2 mm of width of keratinized gingiva [KG] ( Table 2) has been known to improve treatment results (6,12). Hence Miller’s Class I recession defects with at least 2 mm of KG yield satisfactory results with GTR technique ( Table 4).

- Recession depth [RD] and Percentage [%] of Root Coverage: At the end of six months, both Group A and B showed statistically significant improvement in RD ( Table 2). This shows that both the treatment procedures can be used successfully for recession coverage. Group A resulted in a mean RD of 0.33 ± 0.45mm and %RC 84.47 ±21.07%. The results are similar to Romagna- Genon C [2001] (3) and Wang H-L *et al* [2001] (5), Trombelli L *et al* [1998] (13), Cetiner D *et al* [2003] (14). Better results were obtained by many studies (15-19). This can be explained by the fact that in the above studies deep and Class II recession defects were used. Deeper defects result in more %RC as compared to shallow defects (20). In the present study, shallow defects were used for the purpose of comparison with Group B, to access, whether the use of GTR is justified instead of SCTG, to avoid second surgical site. At the end of six months a mean %RC of 81.67±22.31% was obtained in Group B and a mean RD of 0.40±0.47 mm. The results are similar to Romagna- Genon C [2001] (3) and Wang H-L *et al* [2001] (5). When Groups A and B were compared, no significant difference was found at the end of six months (T Table 3). Cetiner D *et al* [2003] (14) evaluated the comparison of connective tissue graft & GTR over a period of 12 months. They found no significant difference between the two groups at the end of 12 months. Scabbia A *et al* [1998] (21) evaluated long term stability of mucogingival complex for GTR. They concluded that clinical outcome achieved following GTR procedure in gingival recession defects can be maintained over a period of 4 years.

- Keratinized Gingiva [KG]: The width of keratinized gingiva increased in both the treatment groups ( Table 2). When both groups were compared after six months of treatment, there was no statistically significant difference between the mean increase in KG [0.13 ± 0.35 mm; p value>0.05]. This result is similar to that obtained by Ro-magna- Genon C [2001] (3) and Wang H-L *et al* [2001] (5). Various biological determinants are implicated in the alteration of gingival dimensions that occur following mucogingival surgery, including induced differentiation of the gingival epithelium by morphogenetic stimuli from the underlying connective tissue, intrinsic specialization resting within the basal cells of the epithelium and post surgery reversal of the mucogingival junction towards its genetically determined location (13). Conversely, in GTR procedures, a moderate increase in width of keratinized tissue derives from the proliferation of granulation tissue from the periodontal, which is able to induce keratinization (18).

- Clinical Attachment Level [CAL], Width Of Attached Gingiva [AG], Residual Probing Depth [RPD]: Both treatments resulted in increase in CAL and AG at the end of six months ( Table 2). When Groups A and B were compared, there was significant difference with more increase in AG and gain in CAL in Group B ( Table 3). This can be correlated to the highly significant difference in RPD with significant increase in RPD in Group A and decrease in RPD in Group B ( Table 2). However in Group B the difference in RPD is not significant. These findings suggest formation of a new attachment on a portion of the covered root surface. Considering the limitation of this study of being only clinical, it is not possible to state whether this gain in attachment in Group B is facilitated by formation of a new attachment. On histological evaluation of healing of SCTG (24), at 60 days long junctional epithelium with no new bone or cementum formation was found. Another histologic case report suggested that various types of tissue attachments including periodontal regeneration may occur over a recession defect after placement of SCTG (25). Whereas histologic evaluation of healing after GTR in other case reports have reported the possibility of obtaining new connective tissue attachment, crestal bone regeneration in the treatment of human buccal recession (26-28).

An interesting observation in this study was that subjects viewed the two techniques as equivalent in terms of outcomes and overall satisfaction ( Table 5). Subjects reported greater overall satisfaction with the Group B, possibly explained elimination of the need for a second surgical procedure and reduction of treatment time.

In conclusion, the purpose of this randomized control trial was to compare the clinical outcomes of traditional subepithelial connective tissue graft [SCTG] versus a guided tissue regeneration [GTR] technique using a double layered collagen membrane for the treatment of shallow Miller’s Class I gingival recessions. Results obtained from this study indicate that both SCTG and GTR can be successfully used to treat recession defects. A GTR technique could offer several advantages over SCTG including elimination of need for a second surgical site for harvesting grafts and related morbidity, reduced surgical time, reduced post surgical discomfort and increase in acceptance of the procedure by the patient.

## References

[1] Wang H L, Al-Shammari KF (2002). Guided tissue regeneration- based root coverage utilizing collagen membranes: Technique and case reports. Classics of Quintessence International.

[2] Langer B, Langer L (1985). Subepithelial connective tissue graft for root coverage. J Periodontol.

[3] Romagna-Genon C (2001). Comparative clinical study of guided tissue regeneration with a bioabsorbable bilayer collagen membrane and subepithelial connective tissue graft. J Periodontol.

[4] 51302Randall HJ (2002). Connective tissue graft combined either double pedicle grafts or coronally positioned pedicle grafts: Results of 266 consecutively treated defects in 200 patients. Int J Periodontics Restorative Dent.

[5] Wang H L, Bunyaratavej P, Labadie M, Shyr Y, MacNeil RL (2001). Comparison of 2 techniques for treatment of gingival recession. J Periodontol.

[6] Borghetti A, Glise JM, Monnet-Corti V, Dejou J (1999). Comparative clinical study of a bioresorbable membrane and subepithelial connective tissue graft in treatment of human gingival recession. J Periodontol.

[7] Muller HP, Stahl M, Eger T (2000). Dynamics of mucosal dimensions after root coverage with a bioresorbable membrane. J Clin Periodontol.

[8] Shieh A T, Wang H L, O'Neil R, Glickman GN, MacNeil RL (1997). Development and clinical evaluation of a root coverage procedure using a collagen barrier membrane. J Periodontol.

[9] Nelson SW (1987). The subpedicle connective tissue graft. A bilaminar reconstructive procedure for the coverage of denuded root surfaces. J Periodontol.

[10] Bunyaratavej P, Wang H L (2001). Collagen Membrane: A review. J Periodontol.

[11] Muller HP, Eger T, Schorb A (1998). Gingival dimensions after root coverage with free connective tissue grafts. J Clin Periodontol.

[12] Rasperini G, Acunzo R, Limiroli E (2011). Decision Making in gingival recession treatment: Scientific evidence and clinical experience. Clinical Advances in Periodontics.

[13] Trombelli L, Scabbia A, Tatakis DN, Calura G (1998). Subpedicle connective tissue graft versus guided tissue regeneration with bioabsorbable membrane in the treatment of human gingival recession defects. J Periodontol.

[14] Cetiner D, Parlar A, Balos K, Alpar R (2003). Comparative clinical study of connective tissue grafts and two types of bioabsorbable barriers in the treatment of localized gingival recessions. J Periodontol.

[15] Bouchard P, Etienne D, Ouhayoun JP, Nilveus R (1994). Subepithelial connective tissue grafts in the treatment of gingival recessions. A comparative study of 2 procedures. J Periodontol.

[16] Randall HJ (1998). A Comparison of 2 Root Coverage Techniques: Guided tissue regeneration with a bioabsorbable matrix style membrane versus a connective tissue graft combined with a coronally positioned pedicle graft without vertical incisions. Results of a series of consecutive cases. J Periodontol.

[17] Cordioli G, Mortarino C, Chierico A, Grusovin MG (2001). , Majzoub Z. Comparison of 2 techniques of subepithelial connective tissue graft in the treatment of gingival recession. J Periodontol.

[18] Paolantonio M (2002). Treatment of gingival recessions by combined periodontal regenerative technique, guided tissue regeneration, subepithelial connective tissue graft. A comparative clinical study. J Periodontol.

[19] Cortellini P, Tonetti M, Baldi C (2009). et al. Does placement of a connective tissue graft improve the outcomes of coronally advanced flap for coverage of single gingival recessions in upper anterior teeth? A multicentre randomized, double blind, clinical trial. J Clin Periodontol.

[20] Deniel A, Cheru R (1990). Treatment of localized gingival recession with subepithelial connective tissue graft and free gingival auto graft. A comparative clinical evaluation. Journal of Indian Dental Assocoation.

[21] Scabbia A, Trombelli L (1998). Long-term stability of the mucogingival complex following guided tissue regeneration in gingival recession defects. J Clin Periodontol.

[22] Roccuzzo M, Lungo M, Corrente G, Gandolfo S (1996). Comparative study of a bioresorbable and a non-resorbable membrane in the treatment of human buccal gingival recessions. J Periodontol.

[23] Tatakis DN, Trombelli L (2000). Gingival recession treatment: Guided tissue regeneration with Bioabsorbable membrane versus connective tissue graft. J Periodontol.

[24] Guiha R, El Khodeiry S, Mota L, Caffesse R (2001). Histological evaluation of healing and revascularization of the subepithelial connective tissue graft. J Periodontol.

[25] Bruno JF, Bowers GM (2000). Histology of human biopsy section following the placement of a subepithelial connective tissue graft. Int J Periodontics Restorative Dent.

[26] Tinti C, Vincenzi G, Cortellini P, Pini Prato G, Clauser C (1992). Guided tissue regeneration in treatment of human facial recession. A 12-case report. J Periodontol.

[27] Cortellini P, Clauser C, Pini Prato G (1993). Histologic assessment of new attachment following the treatment of a human buccal recession by means of a guided tissue regeneration procedure. J Periodontol.

[28] Waterman CA (1997). Guided tissue regeneration using a bioabsorbable membrane in the treatment of human buccal recession. A re-entry study. J Periodontol.

